# Discussion of Average versus Extreme Case Severity in Pandemic Risk Communications

**DOI:** 10.3201/eid2304.161600

**Published:** 2017-04

**Authors:** Brian J. Zikmund-Fisher, Aaron M. Scherer, Megan Knaus, Enny Das, Angela Fagerlin

**Affiliations:** University of Michigan, Ann Arbor, Michigan, USA (B.J. Zikmund-Fisher, A.M. Scherer, M. Knaus);; University of Iowa, Iowa City, Iowa, USA (A.M. Scherer);; Radboud University Nijmegen, Nijmegen, The Netherlands (E. Das);; University of Utah, Salt Lake City, Utah, USA (A. Fagerlin);; Salt Lake City VA Center for Informatics Decision Enhancement and Surveillance, Salt Lake City (A. Fagerlin)

**Keywords:** risk communication, health education, influenza, pandemic, viruses, surveys, the Netherlands

## Abstract

To investigate determinants of the public’s perceptions of disease threat, in 2015 we conducted a randomized survey experiment in the Netherlands. Adults who read a mock news article describing average +or extreme outcomes from a hypothetical influenza pandemic were more influenced by average than by extreme case information. Presenting both types of information simultaneously appeared counterproductive.

When pandemics strike, clear and timely communication is essential to raising public awareness of disease threat and motivating preventive behaviors (*1*). Yet, in most pandemics, the experience of affected persons is heterogeneous: a subset of persons have severe symptoms or sequelae, whereas most affected persons have much milder symptoms or sequelae. This heterogeneity creates a dilemma: Should communications about new infectious disease threats emphasize the character and severity of modal cases, which represents what most persons will experience, or should they focus on the severity of extreme cases to make clear the potential threat, even if that threat is highly unlikely? Both types of information are clearly important. Yet, risk messages are inherently difficult to understand, and providing multiple types of information simultaneously might undermine the public’s understanding of a threat. Simplicity of message enables communications to stick with target audiences, and limiting communications to fewer, clearly contextualized, issues can increase efficacy (*2*,*3*).

To begin to address this communications dilemma, during 2015 we conducted a randomized survey experiment with adult residents of the Netherlands who participate in an online panel administered by Survey Sampling International (https://www.surveysampling.com/). We established quotas for age and sex that approximated the distributions of these characteristics in the population of the Netherlands (Technical Appendix). Upon completing the survey, participants received modest prizes.

Participants read a mock news article about a new pandemic (referred to as H7N3 influenza) spreading within the Netherlands. We randomly varied how the article discussed the average case severity, which was 1) not discussed, 2) described as mild (moderate fever and cough; generally goes away by itself), or 3) described as moderately severe (high fever, cough, vomiting; generally requires intravenous medication and hospitalization). We also independently varied the description of extreme cases, which were 1) not discussed, 2) described as (relatively) mild (requiring 1–2 days of hospitalization because of difficulty breathing, dizziness, and persistent coughing), or 3) described as moderately severe (requiring hospitalization [and causing 1 death] because of difficulty breathing, dizziness, severe coughing, and fluid in the lungs). This randomization resulted in a 3 × 3 between-subjects factorial design. Following guidelines for effective health messages (*4*), all articles included a (fixed) efficacy message, instructing readers to cover their mouths for coughs and sneezes and wash hands frequently to prevent disease spread (online Technical Appendix). This design received exempt status approval from the University of Michigan Medical Institutional Review Board (Ann Arbor, MI, USA).

Our analyses focused on 3 questions: how much respondents would worry if symptoms developed, how much they would worry about extreme effects if they contracted the disease, and participants’ vaccination intentions if a vaccine were available. All questions were 5-point Likert scales, where higher values represented greater worry or intent to vaccinate. Although absolute rates of concern and vaccination intentions are not generalizable from the hypothetical scenario, significant differences among the experimental conditions should be. We conducted 3 × 3 analyses of variance and ordered logistic regression analyses of each outcome with variables for each level of average and/or extreme case information (not present, mild, moderate). The results showed close correspondence, so for simplicity we report only analysis of variance results.

A total of 2,695 participants completed the survey and answered the 3 primary outcome questions. Average age was 49.2 (SD ± 15.6; range 18–96) years, and 49.8% of respondents reported being female.

Overall, respondents were most sensitive to descriptions of average case severity: worry if symptoms: F(2,2686) = 20.87, p<0.001; worry about extreme: F(2,2686) = 6.16, p = 0.002; vaccination intentions: F(2,2686) = 7.56, p<0.001. By contrast, the main effect of extreme case information was nonsignificant in all 3 analyses (0.16<p<0.77). However, we noticed evidence of an interaction effect for vaccination intentions (F[2,2686] = 3.23, p = 0.01).

The main effect of average case information was clearly visible among respondents receiving no information about extreme cases (Table, first column). Yet, the effect of average case information appears muted (less variance) when extreme case descriptions were also presented. In fact, if participants were told that the average case was moderately severe (Table, bottom row), adding extreme case information (either severity level) did not increase worry or vaccination intentions, and the trend is negative.

Our data suggest that information about average cases and extreme cases did not have additive effects on participants’ responses. We observed the strongest effects (positive and negative) of average case information when information about extreme cases was not provided. Providing average case information might inhibit consideration of just how serious the disease could be. Average case information also might have higher personal relevance to the public because extreme cases are more easily discounted. If so, public health communications about new threats should avoid presenting both types of information simultaneously.

**Table:** Differences in ratings of worry and vaccination intentions compared with ratings when no information was provided about a hypothetical influenza pandemic, the Netherlands, 2015*

Average case scenario	Extreme case scenario		
	No Information	Mild severity	Moderate severity
No information			
Worry if symptoms	Reference	–0.01	+0.20
Worry about extreme	Reference	+0.07	+0.13
Vaccination intentions	Reference	–0.16	+0.18
Mild severity			
Worry if symptoms	–0.23	–0.08	–0.07
Worry about extreme	–0.07	–0.01	+0.01
Vaccination intentions	–0.25	–0.18	–0.12
Moderate severity			
Worry if symptoms	+0.26	+0.18	+0.17
Worry about extreme	+0.22	+0.13	+0.13
Vaccination intentions	+0.21	+0.05	–0.06

+, increased worry; –, decreased worry.

Technical Appendix Participants, scenarios, and limitations of a survey about a hypothetical influenza pandemic, the Netherlands, 2015.
