# Supplementary material for: Discussion of Average versus Extreme Case Severity in Pandemic Risk Communications
Source: Emerg Infect Dis. 2017 Apr;23(4):706–8. doi: 10.3201/eid2304.161600 (PMC5367401; doi:10.3201/eid2304.161600)
Supplement: Technical Appendix — Participants, scenarios, and limitations of a survey about a hypothetical influenza pandemic, the Netherlands, 2015. [file 16-1600-Techapp-s1.pdf]

Article DOI: http://dx.doi.org/10.3201/eid2304.161600

Discussion of Average versus Extreme Case Severity in Pandemic Risk Communications

Technical Appendix

Participants

Participants were members of an online panel maintained by the survey research company Survey Sampling International (SSI, https://www.surveysampling.com/). Panel members were originally recruited by a variety of opt-in methods, including online banners, TV ads, emails, apps, social media influencers, and websites. All SSI survey participants go through SSI's rigorous quality controls within the SSI Dynamix sampling platform before being included in any sample. At the time of this survey (April 2015), SSI's panel in the Netherlands had ≈107,000 members, drawn from all parts of the country in roughly similar proportions to the overall population distribution.

Sampling occurred as follows: Whenever an individual panel member logged on to SSI's system and declared his or her availability to take a survey, he or she was then randomized by a dynamic algorithm to 1 of the multiple surveys available at that time. This randomization algorithm adjusted the likelihood of assigning a respondent to a particular survey on the basis of respondent demographic characteristics and the needs of the currently fielded surveys. We established age (3 categories) and sex quotas (all combinations), and randomization continued until the total number of completes and all subquotas were achieved. Given the importance of communicating about pandemic influenza to older populations, our age quotas specifically included a minimum number of older adults.

Scenarios

Once directed to our survey, participants were randomized to view 1 of 9 scenarios, which varied the level of information included in the mock news article regarding average case information and extreme case information. The specific language is as follows:

Average Case Information

- No information
- Mild level information: Symptoms generally go away without medicine. Most of those who have gotten sick have experienced moderate fever with cough and body aches.
- Moderately severe level information: Symptoms generally go away with the help of intravenous medication that requires hospitalization. Most of those who have gotten sick have experienced high fever with cough, body aches, and nausea with severe vomiting.

Extreme Case Information

- No information
- Mild level information: Some extreme cases required evaluation by a doctor and 1–2 days of hospitalization. These individuals experienced difficulty breathing, sudden dizziness, and severe persistent coughing. If you are experiencing any of these symptoms, you should seek medical care immediately.
- Moderately severe level information: Some extreme cases required hospitalization, and in 1 case, the person died due to lung failure. The individuals who were hospitalized experienced difficulty breathing, sudden dizziness, severe persistent coughing, and fluid in the lungs requiring breathing tubes. If you are experiencing any of these symptoms, you should seek medical care immediately.

Illustrative English-language examples of each of the 9 scenarios follow. All study materials were translated into Dutch and then back-translated into English to check for consistency. Survey respondents all received the Dutch versions of the scenarios and questions.

Limitations

Our study is primarily limited by its use of an Internet sample from a single European country. Internet samples are by definition nonrepresentative in that participants have volunteered to take surveys. In addition, although we used age and sex quotas to ensure at least a moderate degree of demographic diversity among study respondents, the final study population differs from the adult population of the Netherlands in both measured and unmeasured ways. Nonetheless, our between-subjects randomized design ensures internal validity of the conclusions. A separate limitation is our use of a hypothetical scenario and disease, which may have affected the degree that participants were able to engage with the materials and consider them in the same way that they would in real life with an actual epidemic. As a result, we present these results as proof of a potentially important issue in pandemic communications but one that needs further studies to confirm its findings and demonstrate its generalizability across situations and populations.

Scenario 1. Neither Average Cases nor Extreme Cases Described (Control)

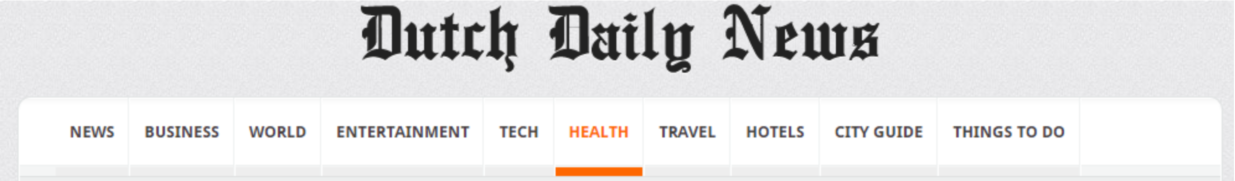

RIVM Reports Rise of H7N3 in the Netherlands

By Jan Brouwer

The number of confirmed cases of H7N3 in the Netherlands has risen to 279 according to health officials at the Rijksinstituut voor Volksgezondheid en Milieu (RIVM).

Dr. Van Leeuwen, an expert with the RIVM, highlighted the number of cases is likely much smaller than the actual number of cases, as there may be many people who have it but who have not seen a doctor or did not have their strain of flu tested. “H7N3 is quickly spreading across the Netherlands and is in every province,” says Dr. Van Leeuwen. The concern is that H7N3 will spread from the Netherlands to other countries in Europe.

Outbreaks of flu viruses like this happen routinely and are closely monitored by health officials. But this rise in H7N3 has drawn considerable attention from health officials and media outlets.

Dr. Leeuwen stressed that RIVM will be closely monitoring cases of H7N3 and will report any changes. In the meantime, RIVM recommends that people can protect themselves by covering their mouth when coughing and sneezing and frequently washing their hands.

Scenario 2. Average Cases Described as Mild, Extreme Cases Absent

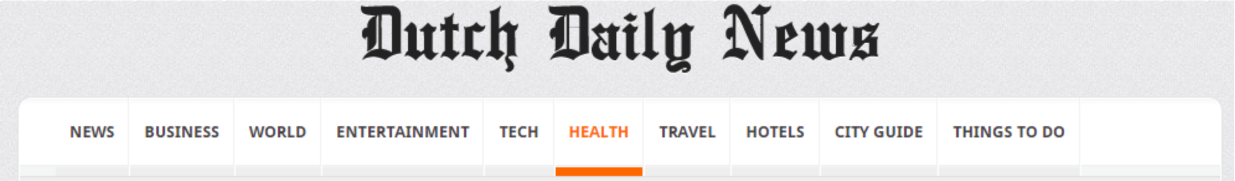

RIVM Reports Rise of H7N3 in the Netherlands

By Jan Brouwer

The number of confirmed cases of H7N3 in the Netherlands has risen to 279 according to health officials at the Rijksinstituut voor Volksgezondheid en Milieu (RIVM).

Dr. Van Leeuwen, an expert with the RIVM, highlighted the number of cases is likely much smaller than the actual number of cases, as there may be many people who have it but who have not seen a doctor or did not have their strain of flu tested. “H7N3 is quickly spreading across the Netherlands and is in every province,” says Dr. Van Leeuwen. The concern is that H7N3 will spread from the Netherlands to other countries in Europe.

Symptoms generally go away without medicine. Most of those who have gotten sick have experienced moderate fever with cough and body aches.

Outbreaks of flu viruses like this happen routinely and are closely monitored by health officials. But this rise in H7N3 has drawn considerable attention from health officials and media outlets.

Dr. Leeuwen stressed that RIVM will be closely monitoring cases of H7N3 and will report any changes. In the meantime, RIVM recommends that people can protect themselves by covering their mouth when coughing and sneezing and frequently washing their hands.

Scenario 3. Average Cases Described as Moderately Severe, Extreme Cases Absent

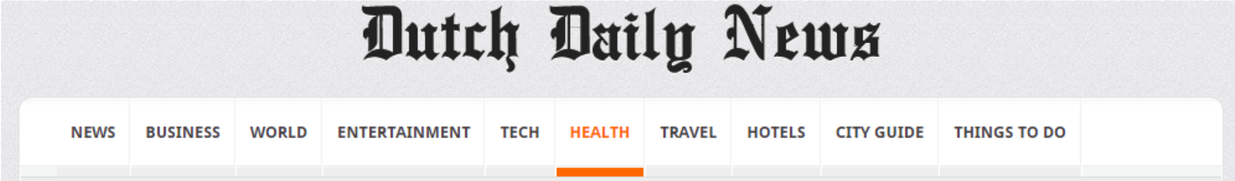

RIVM Reports Rise of H7N3 in the Netherlands

By Jan Brouwer

The number of confirmed cases of H7N3 in the Netherlands has risen to 279 according to health officials at the Rijksinstituut voor Volksgezondheid en Milieu (RIVM).

Dr. Van Leeuwen, an expert with the RIVM, highlighted the number of cases is likely much smaller than the actual number of cases, as there may be many people who have it but who have not seen a doctor or did not have their strain of flu tested. “H7N3 is quickly spreading across the Netherlands and is in every province,” says Dr. Van Leeuwen. The concern is that H7N3 will spread from the Netherlands to other countries in Europe.

Symptoms generally go away with the help of IV medication that requires hospitalization. Most of those who have gotten sick have experienced high fever with cough, body aches, and nausea with severe vomiting.

Outbreaks of flu viruses like this happen routinely and are closely monitored by health officials. But this rise in H7N3 has drawn considerable attention from health officials and media outlets.

Dr. Leeuwen stressed that RIVM will be closely monitoring cases of H7N3 and will report any changes. In the meantime, RIVM recommends that people can protect themselves by covering their mouth when coughing and sneezing and frequently washing their hands.

Scenario 4. Average Cases Absent, Extreme Cases Described as Mild

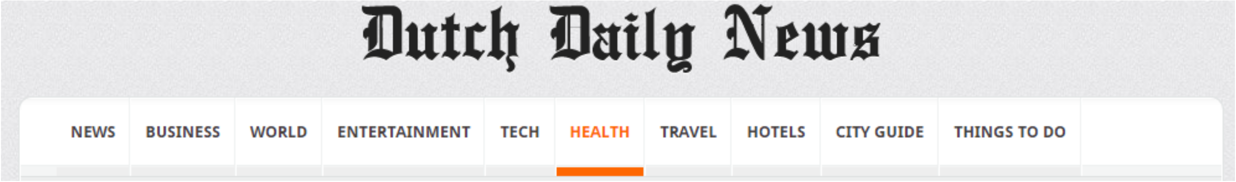

RIVM Reports Rise of H7N3 in the Netherlands

By Jan Brouwer

The number of confirmed cases of H7N3 in the Netherlands has risen to 279 according to health officials at the Rijksinstituut voor Volksgezondheid en Milieu (RIVM).

Dr. Van Leeuwen, an expert with the RIVM, highlighted the number of cases is likely much smaller than the actual number of cases, as there may be many people who have it but who have not seen a doctor or did not have their strain of flu tested. "H7N3 is quickly spreading across the Netherlands and is in every province," says Dr. Van Leeuwen. The concern is that H7N3 will spread from the Netherlands to other countries in Europe.

Some extreme cases required evaluation by a doctor and 1–2 days of hospitalization. These individuals experienced difficulty breathing, sudden dizziness, and severe persistent coughing. If you are experiencing any of these symptoms, you should seek medical care immediately.

Outbreaks of flu viruses like this happen routinely and are closely monitored by health officials. But this rise in H7N3 has drawn considerable attention from health officials and media outlets.

Dr. Leeuwen stressed that RIVM will be closely monitoring cases of H7N3 and will report any changes. In the meantime, RIVM recommends that people can protect themselves by covering their mouth when coughing and sneezing and frequently washing their hands.

Scenario 5. Average Cases Absent, Extreme Cases Described as Moderately Severe

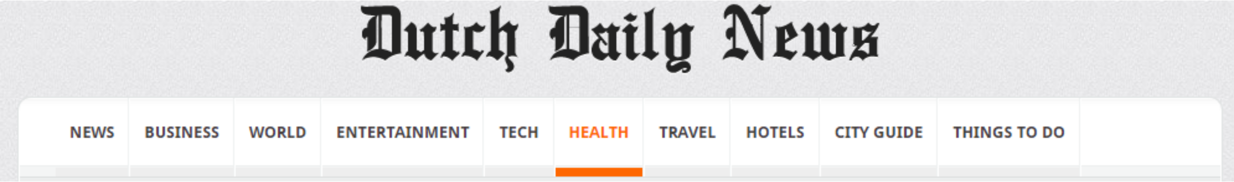

RIVM Reports Rise of H7N3 in the Netherlands

By Jan Brouwer

The number of confirmed cases of H7N3 in the Netherlands has risen to 279 according to health officials at the Rijksinstituut voor Volksgezondheid en Milieu (RIVM).

Dr. Van Leeuwen, an expert with the RIVM, highlighted the number of cases is likely much smaller than the actual number of cases, as there may be many people who have it but who have not seen a doctor or did not have their strain of flu tested. “H7N3 is quickly spreading across the Netherlands and is in every province,” says Dr. Van Leeuwen. The concern is that H7N3 will spread from the Netherlands to other countries in Europe.

Some extreme cases required hospitalization, and in one case, the person died due to lung failure. The individuals who were hospitalized experienced difficulty breathing, sudden dizziness, severe persistent coughing, and fluid in the lungs requiring breathing tubes. If you are experiencing any of these symptoms, you should seek medical care immediately.

Outbreaks of flu viruses like this happen routinely and are closely monitored by health officials. But this rise in H7N3 has drawn considerable attention from health officials and media outlets.

Dr. Leeuwen stressed that RIVM will be closely monitoring cases of H7N3 and will report any changes. In the meantime, RIVM recommends that people can protect themselves by covering their mouth when coughing and sneezing and frequently washing their hands.

Scenario 6. Average Cases and Extreme Cases Both Described as Mild

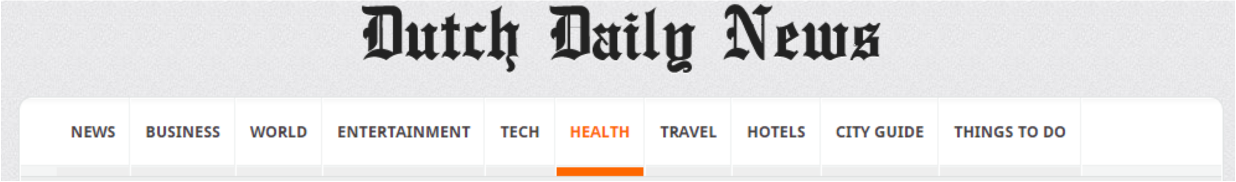

RIVM Reports Rise of H7N3 in the Netherlands

By Jan Brouwer

The number of confirmed cases of H7N3 in the Netherlands has risen to 279 according to health officials at the Rijksinstituut voor Volksgezondheid en Milieu (RIVM).

Dr. Van Leeuwen, an expert with the RIVM, highlighted the number of cases is likely much smaller than the actual number of cases, as there may be many people who have it but who have not seen a doctor or did not have their strain of flu tested. “H7N3 is quickly spreading across the Netherlands and is in every province,” says Dr. Van Leeuwen. The concern is that H7N3 will spread from the Netherlands to other countries in Europe.

Symptoms generally go away without medicine. Most of those who have gotten sick have experienced moderate fever with cough and body aches.

Some extreme cases required evaluation by a doctor and 1–2 days of hospitalization. These individuals experienced difficulty breathing, sudden dizziness, and severe persistent coughing. If you are experiencing any of these symptoms, you should seek medical care immediately.

Outbreaks of flu viruses like this happen routinely and are closely monitored by health officials. But this rise in H7N3 has drawn considerable attention from health officials and media outlets.

Dr. Leeuwen stressed that RIVM will be closely monitoring cases of H7N3 and will report any changes. In the meantime, RIVM recommends that people can protect themselves by covering their mouth when coughing and sneezing and frequently washing their hands.

Scenario 7. Average Cases Described as Mild, Extreme Cases Described as Moderately Severe

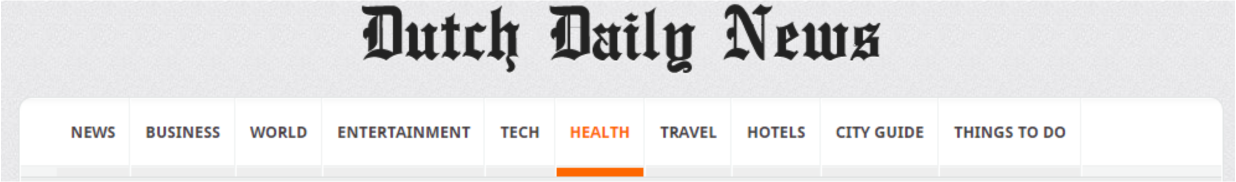

RIVM Reports Rise of H7N3 in the Netherlands

By Jan Brouwer

The number of confirmed cases of H7N3 in the Netherlands has risen to 279 according to health officials at the Rijksinstituut voor Volksgezondheid en Milieu (RIVM).

Dr. Van Leeuwen, an expert with the RIVM, highlighted the number of cases is likely much smaller than the actual number of cases, as there may be many people who have it but who have not seen a doctor or did not have their strain of flu tested. “H7N3 is quickly spreading across the Netherlands and is in every province,” says Dr. Van Leeuwen. The concern is that H7N3 will spread from the Netherlands to other countries in Europe.

Symptoms generally go away without medicine. Most of those who have gotten sick have experienced moderate fever with cough and body aches.

Some extreme cases required hospitalization, and in one case, the person died due to lung failure. The individuals who were hospitalized experienced difficulty breathing, sudden dizziness, severe persistent coughing, and fluid in the lungs requiring breathing tubes. If you are experiencing any of these symptoms, you should seek medical care immediately.

Outbreaks of flu viruses like this happen routinely and are closely monitored by health officials. But this rise in H7N3 has drawn considerable attention from health officials and media outlets.

Dr. Leeuwen stressed that RIVM will be closely monitoring cases of H7N3 and will report any changes. In the meantime, RIVM recommends that people can protect themselves by covering their mouth when coughing and sneezing and frequently washing their hands.

Scenario 8. Average Cases Described as Moderately Severe, Extreme Cases Described as Mild

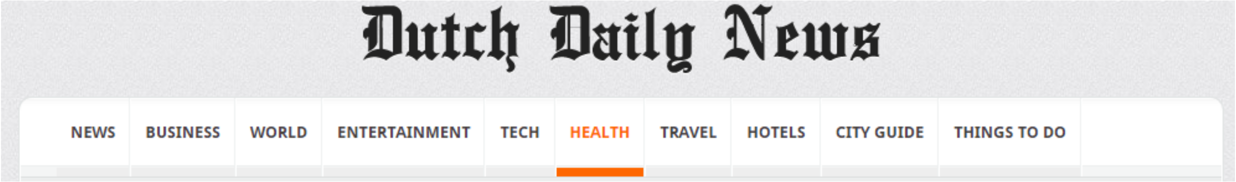

RIVM Reports Rise of H7N3 in the Netherlands

By Jan Brouwer

The number of confirmed cases of H7N3 in the Netherlands has risen to 279 according to health officials at the Rijksinstituut voor Volksgezondheid en Milieu (RIVM).

Dr. Van Leeuwen, an expert with the RIVM, highlighted the number of cases is likely much smaller than the actual number of cases, as there may be many people who have it but who have not seen a doctor or did not have their strain of flu tested. “H7N3 is quickly spreading across the Netherlands and is in every province,” says Dr. Van Leeuwen. The concern is that H7N3 will spread from the Netherlands to other countries in Europe.

Symptoms generally go away with the help of IV medication that requires hospitalization. Most of those who have gotten sick have experienced high fever with cough, body aches, and nausea with severe vomiting.

Some extreme cases required evaluation by a doctor and 1–2 days of hospitalization. These individuals experienced difficulty breathing, sudden dizziness, and severe persistent coughing. If you are experiencing any of these symptoms, you should seek medical care immediately.

Outbreaks of flu viruses like this happen routinely and are closely monitored by health officials. But this rise in H7N3 has drawn considerable attention from health officials and media outlets.

Dr. Leeuwen stressed that RIVM will be closely monitoring cases of H7N3 and will report any changes. In the meantime, RIVM recommends that people can protect themselves by covering their mouth when coughing and sneezing and frequently washing their hands.

Scenario 9. Average Cases and Extreme Cases Described as Moderately Severe

Dutch Daily News

NEWS | BUSINESS | WORLD | ENTERTAINMENT | TECH | HEALTH | TRAVEL | HOTELS | CITY GUIDE | THINGS TO DO

RIVM Reports Rise of H7N3 in the Netherlands

By Jan Brouwer

The number of confirmed cases of H7N3 in the Netherlands has risen to 279 according to health officials at the Rijksinstituut voor Volksgezondheid en Milieu (RIVM).

Dr. Van Leeuwen, an expert with the RIVM, highlighted the number of cases is likely much smaller than the actual number of cases, as there may be many people who have it but who have not seen a doctor or did not have their strain of flu tested. “H7N3 is quickly spreading across the Netherlands and is in every province,” says Dr. Van Leeuwen. The concern is that H7N3 will spread from the Netherlands to other countries in Europe.

Symptoms generally go away with the help of IV medication that requires hospitalization. Most of those who have gotten sick have experienced high fever with cough, body aches, and nausea with severe vomiting.

Some extreme cases required hospitalization, and in one case, the person died due to lung failure. The individuals who were hospitalized experienced difficulty breathing, sudden dizziness, severe persistent coughing, and fluid in the lungs requiring breathing tubes. If you are experiencing any of these symptoms, you should seek medical care immediately.

Outbreaks of flu viruses like this happen routinely and are closely monitored by health officials. But this rise in H7N3 has drawn considerable attention from health officials and media outlets.

Dr. Leeuwen stressed that RIVM will be closely monitoring cases of H7N3 and will report any changes. In the meantime, RIVM recommends that people can protect themselves by covering their mouth when coughing and sneezing and frequently washing their hands.
